# High-Pitch, Low-Voltage and Low-Iodine-Concentration CT Angiography of Aorta: Assessment of Image Quality and Radiation Dose with Iterative Reconstruction

**DOI:** 10.1371/journal.pone.0117469

**Published:** 2015-02-02

**Authors:** Yanguang Shen, Zhonghua Sun, Lei Xu, Yu Li, Nan Zhang, Zixu Yan, Zhanming Fan

**Affiliations:** 1 Department of Radiology, Beijing Anzhen Hospital, Capital Medical University—Beijing Institute of Heart Lung and Blood Vessel Diseases, Beijing, China; 2 Department of Radiology, Hospital Affiliated to Hainan Medical College, Haikou, City of Hainan Province, China; 3 Discipline of Medical Imaging, Department of Imaging and Applied Physics, Curtin University, Perth, Australia; Shenzhen institutes of advanced technology, CHINA

## Abstract

**Objective:**

To assess the image quality of aorta obtained by dual-source computed tomography angiography (DSCTA), performed with high pitch, low tube voltage, and low iodine concentration contrast medium (CM) with images reconstructed using iterative reconstruction (IR).

**Methods:**

One hundred patients randomly allocated to receive one of two types of CM underwent DSCTA with the electrocardiogram-triggered Flash protocol. In the low-iodine group, 50 patients received CM containing 270 mg I/mL and were scanned at low tube voltage (100 kVp). In the high-iodine CM group, 50 patients received CM containing 370 mg I/mL and were scanned at the tube voltage (120 kVp). The filtered back projection (FBP) algorithm was used for reconstruction in both groups. In addition, the IR algorithm was used in the low-iodine group. Image quality of the aorta was analyzed subjectively by a 3-point grading scale and objectively by measuring the CT attenuation in terms of the signal- and contrast-to-noise ratios (SNR and CNR, respectively). Radiation and CM doses were compared.

**Results:**

The CT attenuation, subjective image quality assessment, SNR, and CNR of various aortic regions of interest did not differ significantly between two groups. In the low-iodine group, images reconstructed by FBP and IR demonstrated significant differences in image noise, SNR, and CNR (p<0.05). The low-iodine group resulted in 34.3% less radiation (4.4 ± 0.5 mSv) than the high-iodine group (6.7 ± 0.6 mSv), and 27.3% less iodine weight (20.36 ± 2.65 g) than the high-iodine group (28 ± 1.98 g). Observers exhibited excellent agreement on the aortic image quality scores (κ = 0.904).

**Conclusions:**

CT images of aorta could be obtained within 2 s by using a DSCT Flash protocol with low tube voltage, IR, and low-iodine-concentration CM. Appropriate contrast enhancement was achieved while maintaining good image quality and decreasing the radiation and iodine doses.

## Introduction

Multislice computed tomography angiography (CTA) has become the preferred method to assess aortic diseases [1–6]. However, X-ray radiation and iodine hazards are the major concern associated with CTA, as repeat CTA scans are commonly performed during pre- and postoperative assessments of aortic disease [7–9]. To address these concerns, clinical studies have recently reported the use of the second-generation dual-source computed tomography (DSCT) Flash protocol for dose reduction. This protocol uses a high-pitch acquisition mode, reducing the scan time for the entire aorta to about 2 s and with resultant very low radiation dose [10–11]. High-pitch CTA of the aorta was shown to reduce radiation exposure by 45–50% and to allow the use of less contrast medium (CM) while maintaining vessel attenuation at a diagnostic level [12]. Liu et al. reported that DSCT can provide motion artifact-free imaging of the ascending aorta at a low radiation dose compared to the conventional protocol [7]. However, that study used a CM with a high concentration of iodine (370 mg I/mL).

Reducing the iodine concentration would help to avoid contrast-induced acute kidney injury (CI-AKI) in at-risk patients because the probability of CI-AKI is mainly determined by the amount of delivered iodine [13–14]. Cademartiri et al. reported that under the same injection volume and flow rate, a CM with a low iodine concentration (hereinafter, low-iodine CM) can reduce the iodine burden to patients [15]. However, previous studies found that low-iodine CM was associated with poorer outcomes for vascular attenuation, image quality, and diagnostic accuracy [15–16]. Using a low-tube-voltage technique may help to improve contrast conspicuity in CTA [16–17], but this technique still results in degraded image quality. On the other hand, iterative reconstruction (IR) techniques can be used to reduce image noise and increase the signal- and contrast-to-noise ratios (SNR and CNR, respectively) [18–20]. Zhang et al. reported with the help of IR algorithm techniques, the head-and-neck CTA with diagnostic quality can be adequately achieved with low tube voltage (80 kVp) and low concentration contrast media (270 mg I/mL). This method could be potentially extended to include any part of the body to reduce the ionizing radiation related risks [21].

To the best of our knowledge, no previous report has studied the image quality of the aorta that is obtained by using low-iodine CM and IR during aortic CTA in the Flash Spiral scan mode. Therefore, the goal of this study was to assess the quality of images of the whole aorta obtained by CTA when using a combination of low-iodine CM and scanning with high pitch, low tube voltage, and IR techniques.

## Methods

### Patient population

One-hundred patients (67 males, 33 females) who were referred for noninvasive whole-aortic DSCT angiography were included in this study. Patients had a mean age of 54.3 ± 15.7 years (range: 18–88 years) and a mean body mass index (BMI) of 24.4 ± 2.6 kg/m^2^ (range: 17.7–29.6). Reasons for referral included suspected aortic disease (*n* = 20), postoperative follow-up after thoraco-abdominal vascular surgery (*n* = 25), endovascular aneurysm repair (*n* = 15), endovascular aortic dissection repair (*n* = 30), and follow-up examination of conservatively treated aortic aneurysm (*n* = 10). General exclusion criteria for contrast-enhanced CT were patients with renal insufficiency (serum creatinine > 1.5 mg/dL), BMI > 30 kg/m^2^, history of allergic reaction to CM, untreated hyperthyroidism, and women who were pregnant or nursing. Age, sex, height, and body weight of all patients were recorded for further analysis. The study was approved by Beijing Anzhen Hospital Ethics Committee, and written informed consent was obtained from all patients.

Patients were randomly assigned to one of two groups, according to the iodine concentration of the CM. The 100 patients was first divided into two groups with respect to the range of BMI (BMI≤25 kg/m^2^ and BMI between 25 kg/m^2^ and 30 kg/m^2^), and each group was further divided into subgroups (low-iodine group and high-iodine group). The low-iodine group (*n* = 50) received Iodixanol 270 as CM (270 mg I/mL, GE Healthcare). The high-iodine group (*n* = 50) received Iopamidol 370 as CM (370 mg I/mL, Shanghai Bracco Sine Pharmaceutical Co., Ltd., China).

### CT image acquisition

All studies were performed on a second-generation 128-slice dual-source computed tomography system (SOMATOM Definition Flash, Siemens Healthcare, Forchheim, Germany). All scans were performed in a cranio-caudal direction with a prospective electrocardiogram (ECG)-triggered Flash protocol. Contrast-enhanced scans were performed from the thoracic inlet to the pubic symphysis. All CT imaging data were acquired while the patient held his or her breath in deep inspiration, to eliminate respiratory motion artifacts. Scanning parameters for both groups were as follows: slice collimation of 128 × 0.6 mm with a z-flying focal spot, gantry rotation time of 280 ms, pitch of 3.2, and tube voltage of 100 kV (low-iodine group) or 120 kV (high-iodine group).

CM was injected with an 18- gauge needle through the right antecubital vein by a dual-syringe power injector. A test bolus of 15 mL of CM followed by 30 mL of saline was used to evaluate the scan delay during the acquisition of a series of dynamic low-dose monitoring scans (100 kV, 20 mA for the low-iodine group; 120 kV, 20 mA for the high-iodine group) at the middle of the descending aorta. Regions of interest (ROIs) were placed within the descending aorta to calculate enhancement over time. Monitoring scans (with a temporal resolution of 1 s) began to be acquired 5 s after the start of the injection. The optimal scan delay time was calculated by adding the peak enhancement time from the monitoring scan to 10 s. The actual image was acquired by using 1 mL of CM per kg body weight, followed by 30 mL of saline solution. The injection rate of CM and saline solution was 4 mL/s for all subjects.

### Image reconstruction

In both the low- and high-iodine groups, CTA images were reconstructed by a conventional filtered back projection (FBP) algorithm with a medium smooth kernel designed for cardiac imaging (B26f). In the low-iodine group, images were also reconstructed by a sinogram-affirmed IR algorithm (SAFIRE, Siemens Healthcare) with the corresponding vascular kernel (I26f). With the IR algorithm, five adjustable strength settings (strength 1–5) were available for adaptation of the noise model (SAFIRE). As recommended by manufacturer, a medium strength of 3 was used.

**Fig 1:**
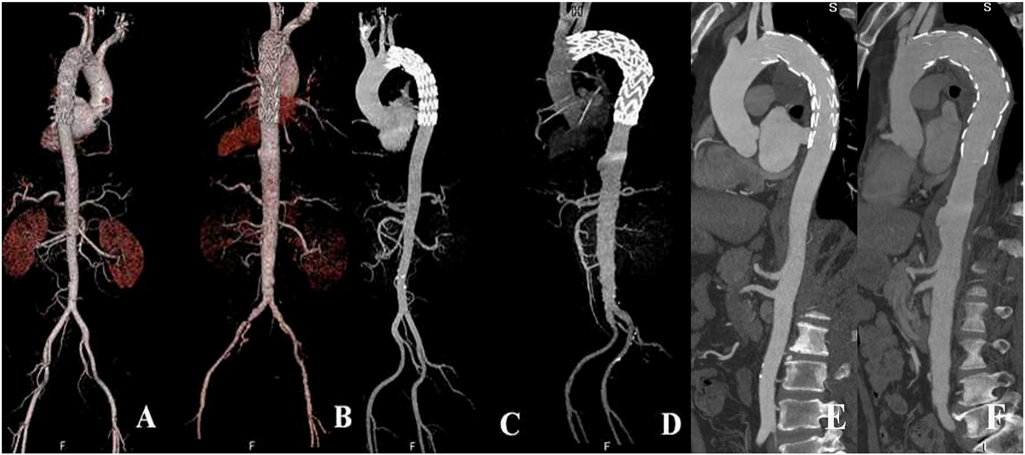
2D and 3D reconstructions with images generated using low-iodine and high-iodine groups. Volume rendering, maximum-intensity projection and multiplanar reformation images (A-F) show endovascular repair of aortic dissection (A, C, E) and aneurysm (B, D, F) with stent graft placed just below the left subclavian artery. A, C and E represent images acquired with the low-iodine protocol, while B, D and F are images generated with the high-iodine protocol. There is no difference in the visualization of stent graft and aortic branches between the two groups.

In both groups, transverse images were reconstructed with a slice thickness of 1 mm in 1-mm increments. Patient information was removed from all images, which were transferred to an external workstation (Syngo Multi-Modality Work Place, CT 2011A, Siemens Healthcare) for further image analysis. Using the axial data, two cardiac radiologists with more than 8 years of experience in cardiac imaging reconstructed the images by volume rendering technique, maximum intensity projection, and multiplanar reconstruction (Fig. 1).

### Evaluation of objective image quality in the aorta

**Fig 2:**
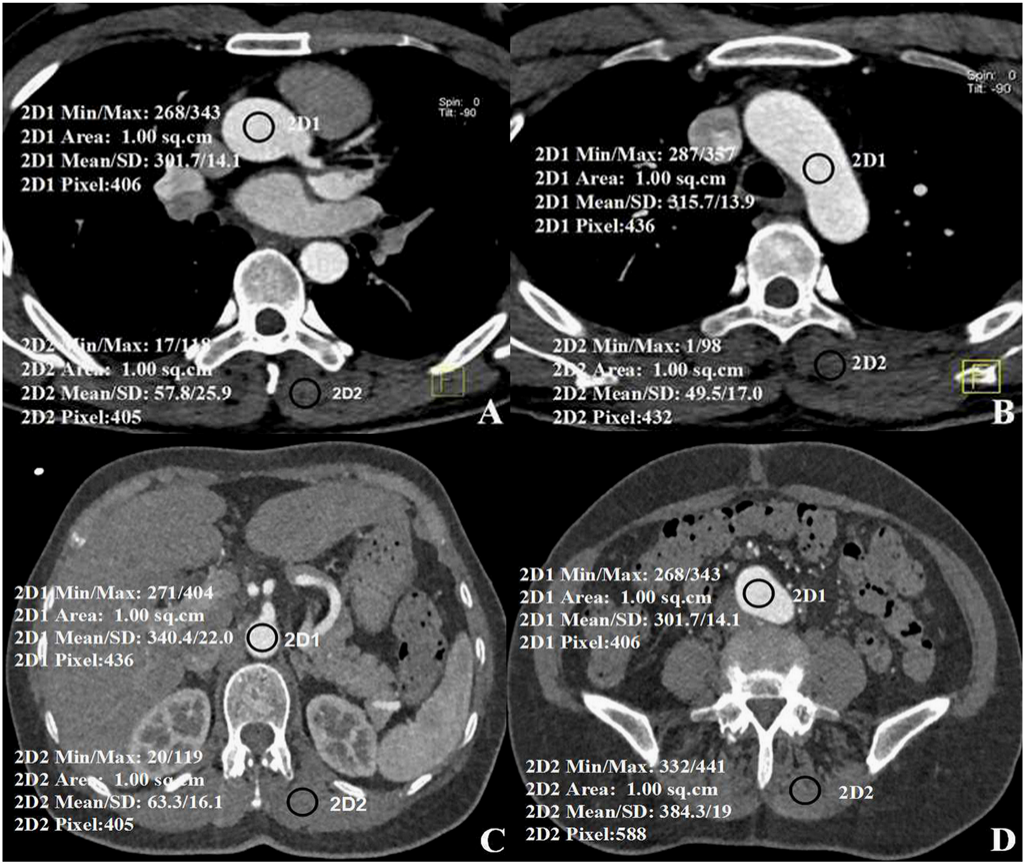
Axial images of aortic segments with paraspinal muscle. A) Lumen of the ascending aortic root, B) the aortic arch, C) the descending aorta at the first lumbar (L1), and D) common iliac artery bifurcation. Mean attenuation values with standard deviations are shown in the images.

**Fig 3:**
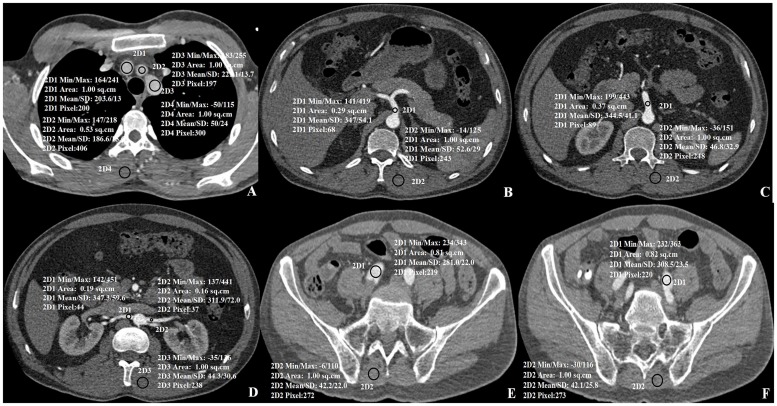
Axial images of aortic branches and segments with paraspinal muscle. A) Lumen of the Brachiocephalic trunk (2D1), the Left common carotid artery (2D2) and the Left subclavian artery (2D3), B) Celiac trunk, C) the superior mesenteric artery, D) the right renal artery (2D1) and the left renal artery (2D2), E) the right common iliac artery, F) the left common iliac artery. Mean attenuation values with standard deviations are shown in the images.

Axial slices were selected to ensure that the aortic enhancement values, aortic image noise, and the same paraspinal muscle noise were measured in identical ROIs in the ascending aortic root, aortic arch, descending aorta at the first lumbar (L1), and iliac artery bifurcation (Fig. 2). Furthermore image noise was also measured in the aortic main branches, including regions in the proximal segment of the brachiocephalic trunk, left common carotid artery, left subclavian artery, celiac trunk, superior mesenteric artery, renal arteries, and the distal segment of the common iliac arteries (Fig. 3). In each patient and for each structure, three ROIs, each measuring 100 mm^2^, were drawn on three consecutive transverse sections. When the vessel area was less than 100 mm^2^, the ROI was drawn to encompass the entire contrast-enhanced aortic lumen. Care was taken to avoid including vessel walls, emboli, calcified plaques, areas of stenosis, or motion artifacts in the ROIs. The mean attenuation (in Hounsfield Unit [HU]) and standard deviation (SD) in each ROI on three consecutive sections were calculated for each target structure (Figs. 2 and 3).

A single reader independently calculated the SNR and CNR in the aortic images. Means and SDs of attenuation of the contrast-enhanced vessel lumen (SI_aorta_ and SD_aorta_, respectively) and the paraspinal muscle tissue (at the same level of the spine in compared images; SI_muscle_ and SD_muscle_, respectively) were recorded. Each parameter was calculated three times, and the mean value was used in the SNR and CNR calculations. Noise ratios were determined by the following equations: SNR = SI_aorta_ – SI_muscle_/SD_aorta_ and CNR = SI_aorta_ – SI_muscle_/SD_muscle_ [10–11,16].

### Assessment of subjective image quality in the aorta

Subjective image quality was independently rated by two radiologists, who had 15 and 10 years of experience in CTA, respectively, and who were blinded to all patient data and to the CM, scanning protocols, and reconstruction algorithms that were used. Images were rated on a 3-point Likert scale, on the basis of contour delineation, presence of motion artifacts, and general image quality (1 = poor, 2 = moderate, and 3 = good) [10]. Grades 2 and 3 were considered diagnostic images.

### Measurement of radiation and CM doses

The CT dose index volume (CTDI_vol_) and dose length product (DLP) were recorded during the scans. The estimated effective dose was derived from the DLP by using the equation Effective dose = DLP × *k*, where *k* is the conversion coefficient for the thoraco-abdominal region (*k* = 0.017 mSvmGy^-1^cm^-1^) [10, 22]. The iodine delivery rate (g I/s) was calculated as follows: Iodine delivery rate = iodine concentration (mg I/mL) × injection rate of CM (mL/s) / 1000 mg/g. The iodine weight (g I) was calculated as follows: Iodine weight = iodine concentration (g I/mL) × injection dose of CM (mL).

### Evaluation of adverse effects due to CM

Patients were asked to rate their discomfort, in terms of pain at the injection site, sensations of cold or heat in the injected vein, and other discomfort, immediately and 15 to 20 min after injections. Scores were reported verbally on a scale from 1 (severe discomfort) to 10 (no discomfort at all) [23]. All adverse effects to both kinds of CM were recorded.

### Statistical analysis

Statistical analysis was performed with the SPSS software package (SPSS V 19.0, Chicago, ILL). Continuous variables were expressed as mean ± SD and were analyzed by independent *t*-tests for normally distributed data or Mann-Whitney U tests for non-normally distributed data. Independent *t*-tests were performed to analyze differences between two groups regarding attenuation values, image noise, and radiation doses. The Mann-Whitney U test was used to detect differences in the subjective evaluation of image quality between the two groups. Cohen’s kappa statistic (κ) was calculated to determine the interreader agreement in the assessment of image quality, where κ > 0.81 indicated excellent, κ = 0.61–0.80 indicated good, κ = 0.41–0.60 indicated moderate, κ = 0.21–0.40 indicated fair, and κ < 0.20 indicated poor agreement. A *P*-value < 0.05 was considered statistically significant for all data analyses.

## Results

### Demographic data and scanning parameters

High-pitch CTA was successfully performed in the 100 patients with suspected aortic disorders. Patient demographics and CTA acquisition characteristics are shown in Table 1. No significant differences were found between groups for any demographic characteristics, scan time, or scan length.

**Table 1:** Patient demographics and characteristics.

Characteristic	Low-iodine group	High-iodine group	*t*-value	*P*-value
No. of patients (male)	50 (39)	50 (38)	——	——
Age (years)	52.66 ± 16.50	55.94 ± 14.96	1.041	0.300
Body weight (kg)	70.33 ± 9.2	70.06 ± 9.73	0.140	0.889
Body height (cm)	170.02 ± 10.21	168.44 ± 6.01	0.963	0.338
BMI^a^ (kg/m^2^)	24.24 ± 2.45	24.63 ± 2.65	-0.761	0.449
Scan time (acquisition time)	1.66 ± 0.09	1.63 ± 0.06	1.521	0.131
Scan length	660.92 ± 41.30	651.31 ± 39.22	1.193	0.236
Tube potential (kVp)	100	120	——	——
Reconstruction algorithm	SAFIRE	FBP	——	——
Reference tube current (mA)	350	350	——	——

No significant differences (*P* > 0.05) were noted between the two groups regarding these demographic data and CTA acquisition characteristics. Abbreviations: BMI, body mass index; SAFIRE, a product of Siemens Healthcare; FBP, filtered back projection.

### Assessment of image quality

**Fig 4:**
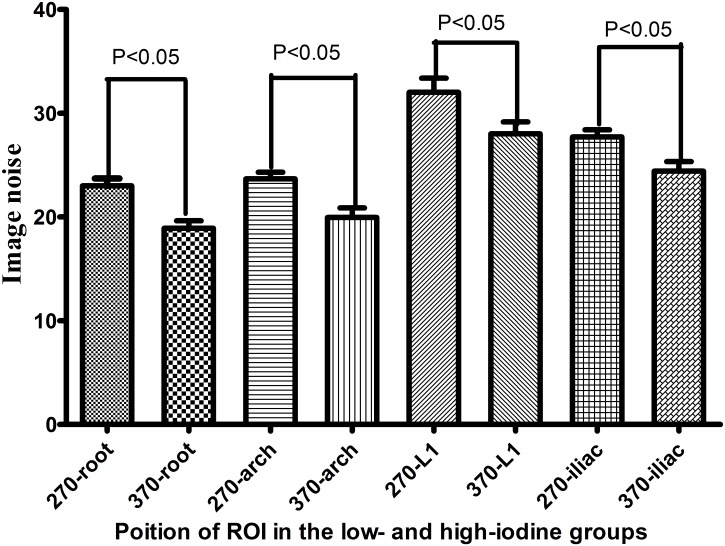
Image noise in aortic segments in the low-iodine and high-iodine groups. Segments include the aorta root, arch, descending aorta at the first lumbar (L1), and the iliac artery bifurcation.

There were no significant differences in mean aortic attenuation, SNR, or CNR between the low- and high-iodine groups of the aorta and aortic branches. However, image noise differed significantly between the groups of the aorta and aortic branches (Tables 2, 3 and Fig. 4). In the low-iodine group, images reconstructed by the FBP and IR algorithms demonstrated significant differences in terms of image noise, SNR, and CNR (*P* < 0.05), but no significant difference in the mean aortic attenuation (Table 4). Of the subgroups by BMI≤25 kg/m^2^, image noise demonstrated significant differences (P < 0.05) in the mean aortic attenuation of ascending aorta root and aorta arch, but no significant difference in SNR, CNR and the mean aortic attenuation of descending aorta at L1and iliac artery bifurcation. No significant difference was found in the subgroups by BMI between 25 kg/m^2^ and 30 kg/m^2^ except the SNR of the ascending aorta root (Table 5). Subjective image quality scores did not differ significantly between the low- and high-iodine groups (2.92 ± 0.27 vs. 2.92 ± 0.40; *P* = 0.917). Interreader agreement was excellent for both groups (κ = 0.904).

**Table 2:** Attenuation, image noise, SNR, and CNR in anatomic regions of interest of aorta.

Item	Low-iodine group (*n* = 50)	High-iodine group (*n* = 50)	*t*-value	*P*-value
Attenuation				
Ascending aorta root	302.85 ± 54.76	320.09 ± 73.16	-1.344	0.185
Aorta arch	309.45 ± 57.96	325.51 ± 69.27	-1.257	0.212
Descending aorta at L1	304.30 ± 60.27	312.54 ± 66.72	-0.648	0.519
Iliac artery bifurcation	308.44 ± 74.08	315.90 ± 59.05	-0.557	0.579
Image noise				
Ascending aorta root	18.90 ± 5.21	23.00 ± 5.00	-4.007	0.001
Aorta arch	19.95 ± 6.40	23.68 ± 4.47	-3.35	0.001
Descending aorta at L1	28.02 ± 8.13	32.03 ± 9.63	-2.250	0.027
Iliac artery bifurcation	24.43 ± 6.30	27.72 ± 4.86	-2.894	0.005
SNR				
Ascending aorta root	14.02 ± 4.02	12.50 ± 3.74	1.959	0.053
Aorta arch	14.18 ± 4.49	12.60 ± 3.91	1.875	0.064
Descending aorta at L1	9.57 ± 3.07	8.96 ± 3.32	0.945	0.347
Iliac artery bifurcation	11.38 ± 4.31	10.19 ± 2.66	1.67	0.98
CNR				
Ascending aorta root	10.88 ± 3.72	10.03 ± 3.28	1.217	0.226
Aorta arch	10.19 ± 2.67	10.10 ± 3.01	0.138	0.890
Descending aorta at L1	11.08 ± 3.51	9.92 ± 3.03	1.772	0.079
Iliac artery bifurcation	11.57 ± 4.09	10.29 ± 3.67	1.658	0.100

SNR, signal-to-noise ratio; CNR, contrast-to-noise ratio.

**Table 3:** Attenuation, image noise, SNR, and CNR in anatomic regions of interest of aortic branches.

Item	Low-iodine group (*n* = 50)	High-iodine group (*n* = 50)	*t*-value	*P*-value
Attenuation				
Brachiocephalic trunk	312.61 ± 60.76	340.86 ± 80.89	-1.975	0.051
Left common carotid artery	328.29 ± 74.33	358.89 ± 84.37	-1.925	0.057
Left subclavian artery	328.31 ± 63.25	358.81 ± 80.89	-1.963	0.053
Celiac trunk	290.03 ± 70.19	300.27 ± 75.26	-0.704	0.483
Superior mesenteric artery	302.81 ± 63.26	308.56 ± 69.60	-0.432	0.667
Right renal artery	282.68 ± 62.96	283.19 ± 71.71	-0.038	0.969
Left renal artery	280.21 ± 73.03	285.29 ± 78.39	-0.335	0.738
Left common iliac artery	281.02 ± 79.45	290.69 ± 64.48	-0.668	0.506
Right common iliac artery	286.41 ± 83.38	295.41 ± 69.57	-0.585-	0.560
Image noise				
Brachiocephalic trunk	19.73 ± 5.72	23.46 ± 5.64	-3.296	0.001
Left common carotid artery	20.97 ± 12.82	25.24 ± 6.96	-2.070	0.041
Left subclavian artery	19.05 ± 5.06	25.61 ± 12.23	-3.509	0.001
Celiac trunk	31.86 ± 9.04	38.06 ± 11.56	-2.991	0.004
Superior mesenteric artery	30.37 ± 8.49	38.29 ± 14.61	-3.310	0.001
Right renal artery	18.90 ± 5.21	23.00 ± 5.00	-2.062	0.042
Left renal artery	36.30 ± 12.64	41.98 ± 12.76	-2.235	0.028
Left common iliac artery	26.66 ± 5.99	30.09 ± 7.82	-2.462	0.016
Right common iliac artery	25.66 ± 6.44	30.18 ± 7.73	-3.177	0.002
SNR				
Brachiocephalic trunk	15.26 ± 4.71	14.25 ± 5.48	0.994	0.332
Left common carotid artery	16.27 ± 5.87	14.13 ± 5.45	1.896	0.061
Left subclavian artery	16.64 ± 5.32	14.56 ± 5.75	1.874	0.064
Celiac trunk	7.93 ± 3.22	7.18 ± 3.17	1.173	0.244
Superior mesenteric artery	8.74 ± 2.75	8.09 ± 6.71	0.632	0.529
Right renal artery	7.99 ± 6.43	6.14 ± 2.23	1.928	0.057
Left renal artery	6.83 ± 3.11	5.86 ± 1.69	1.945	0.055
Left common iliac artery	9.34 ± 3.44	8.87 ± 3.13	0.715	0.476
Right common iliac artery	10.04 ± 4.17	9.03 ± 3.72	1.273	0.206
CNR				
Brachiocephalic trunk	9.67 ± 3.42	10.24 ± 3.18	-0.853	0.396
Left common carotid artery	10.41 ± 3.94	10.87 ± 3.31	-0.640	0.524
Left subclavian artery	10.28 ± 3.89	10.83 ± 3.28	-0.764	0.447
Celiac trunk	8.93 ± 3.62	8.04 ± 2.92	1.349	0.181
Superior mesenteric artery	8.93 ± 3.62	8.04 ± 2.92	1.814	0.073
Right renal artery	8.91 ± 3.53	7.80 ± 2.64	1.776	0.079
Left renal artery	8.79 ± 3.39	7.96 ± 3.31	1.245	0.216
Left common iliac artery	8.50 ± 3.76	8.11 ± 3.11	0.558	0.578
Right common iliac artery	9.18 ± 4.15	8.16 ± 3.46	1.334	0.185

SNR, signal-to-noise ratio; CNR, contrast-to-noise ratio.

**Table 4:** Attenuation, image noise, SNR, and CNR in low-iodine group.

Item	FBP (*n* = 50)	IR (*n* = 50)	*t*-value	*P*-value
Attenuation (HU)				
Ascending aorta root	302.25 ± 55.4	302.85 ± 54.76	-0.054	0.957
Aorta arch	308.50 ± 50.73	309.45 ± 57.96	-0.087	0.931
Descending aorta at L1	299.53 ± 57.68	304.3 ± 60.27	-0.405	0.687
Iliac artery bifurcation	306.78 ± 73.95	308.44 ± 74.08	-0.112	0.911
Image noise				
Ascending aorta root	22.40 ± 4.82	18.90 ± 5.21	3.483	0.001
Aorta arch	23.93 ± 4.99	19.95 ± 6.48	3.435	0.001
Descending aorta at L1	32.24 ± 8.42	28.02 ± 8.10	2.555	0.012
Iliac artery bifurcation	31.02 ± 6.97	24.43 ± 6.40	4.929	0.0001
SNR				
Ascending aorta root	12.00 ± 4.21	14.02 ± 4.02	-2.454	0.016
Aorta arch	11.58 ± 3.77	14.18 ± 4.49	-3.136	0.002
Descending aorta at L1	8.24 ± 3.08	9.57 ± 3.07	-2.153	0.034
Iliac artery bifurcation	8.61 ± 3.18	11.38 ± 4.31	-3.652	0.0001
CNR				
Ascending aorta root	9.39 ± 3.30	10.88 ± 3.72	-2.119	0.037
Aorta arch	7.73 ± 1.57	10.11 ± 3.01	-4.945	0.000
Descending aorta at L1	9.13 ± 3.92	11.08 ± 3.51	-2.621	0.010
Iliac artery bifurcation	9.23 ± 3.24	11.58 ± 4.10	-3.168	0.002

SNR, signal-to-noise ratio; CNR, contrast-to-noise ratio.

**Table 5:** Attenuation, image noise, SNR, and CNR in various regions of aorta in Low-iodine and High-iodine group in patients stratified by BMI.

	BMI ≤ 25 kg/m^2^			BMI 25 kg/m^2^ and 30 kg/m^2^		
	Low-iodine group	High-iodine group	P	Low-iodine group	High-iodine group	P
Patients, n	33	30	—	17	20	—
CT attenuation						
Ascending aorta root	292.71 ± 49.14	329.54 ± 81.02	0.031	322.52 ± 61.08	305.93 ± 58.59	0.406
Aorta arch	296.52 ± 52.02	334.27 ±78.45	0.027	334.56 ± 62.14	312.36 ± 51.6	0.244
Descending aorta at L1	299.19 ± 59.53	321.92 ± 73.61	0.181	314.21 ± 62.28	298.46 ± 53.50	0.413
Iliac artery bifurcation	304.71 ± 67.66	325 ± 61.30	0.208	315.67 ± 86.98	301.53 ± 53.81	0.549
Image noise						
Ascending aorta root	17.51 ± 3.79	21.57 ± 5.04	0.001	21.61 ± 6.54	25.15 ± 4.24	0.055
Aorta arch	18.45 ± 6.51	22.68 ± 4.53	0.004	22.86 ± 5.50	25.19 ± 4.03	0.147
Descending aorta at L1	25.57 ± 6.87	29.86 ± 8.37	0.029	32.79 ± 8.38	35.27 ± 10.66	0.442
Iliac artery bifurcation	23.27 ± 6.21	26.75 ± 5.20	0.020	26.67 ± 6.33	29.17 ± 4.01	0.155
SNR						
Ascending aorta root	14.19 ± 3.80	13.57 ± 4.11	0.537	13.69 ± 4.53	10.90 ± 2.39	0.022
Aorta arch	14.42 ± 4.17	13.48 ± 4.49	0.391	13.71 ± 5.16	11.28 ± 2.38	0.068
Descending aorta at L1	10.08 ± 3.24	9.72 ± 3.33	0.667	8.58 ± 2.52	7.82 ± 3.04	0.425
Iliac artery bifurcation	11.81 ± 4.76	10.86 ± 2.86	0.338	10.55 ± 3.24	9.17 ± 1.99	0.121
CNR						
Ascending aorta root	11.33 ± 4.05	10.46 ± 3.57	0.373	10.01 ± 2.85	9.38 ± 2.74	0.496
Aorta arch	9.90 ± 2.87	10.69 ± 3.05	0.293	10.74 ± 2.23	9.23 ± 2.80	0.082
Descending aorta at L1	11.57 ± 3.56	10.35 ± 3.15	0.158	10.13 ± 3.29	9.27 ± 2.80	0.394
Iliac artery bifurcation	11.79 ± 4.14	11.09 ± 3.77	0.483	11.15 ± 4.10	9.08 ± 3.25	0.095

SNR, signal-to-noise ratio; CNR, contrast-to-noise ratio.

### Estimation of radiation and CM doses

The CTDI_vol_ and DLP were significantly higher in the high-iodine group compared to the low-iodine group (Table 6). A comparison of the effective radiation doses revealed that the low-iodine group received 34.3% less radiation than the high-iodine group (*P* < 0.001). The iodine weight and iodine delivery rate were lower in the low-iodine compared to the high-iodine group (Table 6, *P* < 0.001).

**Table 6:** Radiation dose and contrast medium dose measurements.

Item	Low-iodine group	High-iodine group	*t*-value	*P*-value
CTDI_vol_ (mGy)	3.64 ± 0.34	6.15 ± 3.76	-4.705	0.0001
DLP (mGy/cm)	258.83 ± 29.92	396.33 ± 36.10	-20.736	0.0001
Effective dose (mSv)	4.40 ± 0.51	6.73 ± 0.61	-20.736	0.0001
Iodine weight (g)	20.36 ± 2.65	28 ± 1.98	-17.374	0.0001

CTDI_vol_, CT dose index volume; DLP, dose length product; ED, effective dose.

### Adverse effects with CM injection

There were no cases of contrast extravasation in any patient. In the low-iodine group, severe, moderate, and mild sensations of heat during the scan were reported by 0 (0%), 7 (14%), and 40 (80%) patients, respectively. In the high-iodine group, these values were 20 (40%), 18 (36%), and 12 (24%) patients, respectively. Three patients (6%) in the low-iodine group experienced sensations of cold, compared to no patients in the high-iodine group.

In the low-iodine group, 5 (10%) patients experienced pain. In this group, 2 patients had moderate adverse reactions (rash and vomit), which occurred 1 min and 6–8 h, respectively, after CM injection. All adverse symptoms were resolved appropriately. No delayed adverse reactions occurred in the high-iodine group.

## Discussion

Since the introduction of multidetector CT technology, CTA has become a commonly performed and routine tool to evaluate diseases of the aorta and its major branches [24]. Various techniques and patient-based strategies have focused on reducing the radiation dose that is delivered during aortic CTA. Given that the radiation dose varies as a function of the tube voltage squared, lowering the tube voltage is an important approach to reducing the radiation dose [25]. The results of this study show that high-pitch low-dose, low iodine CTA reconstructed with IR provides diagnostically adequate image quality for evaluating aortic diseases, which is consistent with findings from others using IR reconstruction algorithms [2, 26].

Lowering the tube voltage has the additional advantage of offering higher attenuation levels for iodinated CM at lower X-ray tube voltages because of a greater photoelectric effect and decreased Compton scattering [27]. The inherent attenuation of iodinated CM increases as the tube voltage approaches the K-edge of iodine (33.2 keV) [28–30]. Therefore, lowering the tube voltage may help to improve vascular attenuation when using the same concentration of CM, or when using lower concentrations without increasing the injection rate. This approach offers the possibility of improving vascular attenuation while using low-iodine CM [15–16].

It is generally accepted that improved vascular visualization can be achieved by increasing the injection rate or iodine concentration of the CM [10, 31–32]. However, the results of our study showed that aortic attenuation could be retained by using a low-iodine CM and reduced tube voltage with the same intravenous CM dose and injection rate (4 mL/s). These findings further demonstrate the potential utility of lowering the tube voltage for increasing the vascular CT values. Using low-iodine CM may reduce the iodine flux and dose. In at-risk patients, using low-iodine CM may help to prevent CI-AKI, which is the third most common cause of acute renal failure among hospitalized patients [13–14, 33–34]. Low-iodine CM can be easily delivered to patients, with less patient discomfort. In our study, the mean iodine dose (iodine weight, 20.36 ± 2.65 g in the low-iodine group) was lower than that in a previous study, indicating the feasibility of using low iodine CM in CTA scans of the aorta. Apparently, it is important to obtain high and homogenous enhancement of the arterial tree in aortic CTA and to synchronize the acquisition with the enhancement. The optimization of acquisition timing and contrast medium delivery is essential for vascular assessment and image postprocessing. Due to the inter-individual hemodynamic variability in patients undergoing aortic CTA, the appropriate scan delay may become more critical since the chances for timing errors are increasingly related to the increased speed of z-axis coverage with the use of multi-detector row CT technology. The test bolus technique provides more information on the individual hemodynamic situation of the study subject. The second-generation dual-source CT allows performance of thoraco-abdominal CTA within less than 2 s and therefore the required enhancement plateau phase can be considerably shortened. Moreover, with short data acquisition time, recirculation effects, which contributed to the prolonged enhancement plateau, can be ignored. Due to these factors, the demand on bolus geometry shifted from a long balanced to a compact bolus [35]. In this study, data acquisition began 25–30 s following injection of contrast medium in all patients, and this is based on the calculation of timing of about 15s by test bolus to reach peak enhancement, plus 10 s of further delay after initiation of the bolus injection and 1.5–2 s of scan time. We chose 25–30 seconds as the scan delay in this study on the basis of our prior experience and other results in the literature [36]. CM volume was acquired by using 1 mL of CM per kg body weight. According to reports available in the literature, adequate vascular enhancement is suggested to be equal or above 200 HU [31,35]. In our experience, the cut off level of 200 HU can be achieved in almost every patient, indicating that our results are consistent with others.

Low-tube-voltage CT scanning has some limitations, including increased image noise due to low photon flux [25] and degraded image quality due to the higher susceptibility to beam-hardening artifacts. There is also a noise penalty due to the simplicity of reconstruction by conventional FBP [37]. An IR algorithm can be used to counteract this problem, as IR minimizes the noise effects while maintaining spatial resolution and other image quality properties. Unlike FBP, the IR technique reconstructs CT datasets by fully modeling the system. The reconstruction process is iterative in nature, to overcome the mathematical complexity introduced by the added modeling [19, 37–38]. The IR technique does not assume that the measured signal is free of noise (X-ray or electronic), but rather uses more accurate statistical modeling during the reconstruction process [31, 38–40].

The results of this study showed that it is possible to achieve diagnostic image quality by using a strength level of 3 in SAFIRE with a low tube voltage (100 kVp). In the low-iodine group, using the IR algorithm reduced image noise and improved the SNR and CNR compared to the FBP algorithm. Image noise was slightly higher in the high-iodine group compared to the low-iodine group, although SNR and CNR did not differ significantly between the two groups. These findings may suggest that a SAFIRE strength level of 3 reduces the noise and increases the SNR or CNR. When combined with a low-iodine CM and low tube voltage, IR can effectively suppress image noise and retain image quality. Moreover, the increased image noise does not necessarily result in diminished subjective image quality, because the increased attenuation of the iodine-containing arterial system and the high attenuation difference between the arterial system and surrounding tissues can partially offset the higher image noise.

The major advantage of lowering the tube voltage is to reduce the radiation dose. However, high pitch and IR are also effective approaches that are increasingly used to reduce the radiation dose. Liu et al. and Bolen et al. [7, 39] showed that imaging of the thoraco-abdominal aorta with ECG-triggered high-pitch CTA provided higher quality images of the aortic root and ascending aorta, with sufficient contrast enhancement and decreased estimated radiation dose compared to standard-pitch helical CT. Our results are consistent with these findings, as both qualitative and quantitative assessments of image quality confirmed that the diagnostic images were acquired with the use of this low-dose protocol.

Studies have demonstrated that IR could increase image quality and reduce the effective radiation dose compared to FBP [16, 22–23]. Winklehner et al. reported that raw data-based IR allowed for a dose reduction of more than 50%, while maintaining the quality of body CTA images [40]. For long z-axis ECG-gated CTA of the whole aorta in patients with aortic diseases, the high radiation dose remains a concern. The results of our study show that using the combination of lower tube voltage, high pitch, and IR offers one strategy for maintaining image quality while minimizing radiation exposure during aortic CTA. This approach may be particularly useful in female patients and in patients who require follow-up for aortic aneurysm, aortic stent graft implantation, and other operations [34,39]. In our study, 70% of patients (70/100) required frequent postoperative follow-up examinations of aortic disease, thus, a low-dose CTA protocol is suitable for these patients, particularly for those treated with endovascular repair. Hansen et al in their recent study showed that low-dose CTA with model-based IR in patients undergoing endovascular aneurysm repair results in up to 73% dose reduction compared to standard CTA protocol (mean dose: 4.4 and 2.4 mSv vs 16.2 and 6.7 mSv, respectively corresponding to the arterial and delayed phases) [26]. The mean effective dose of our low-dose protocol is 4.4 mSv, which is the same as that reported in Hansen’s study as only arterial phase was involved in our scans. Koike et al. showed that low-dose dynamic volumetric CTA is feasible after endovascular aneurysm repair [6]. However, the mean effective dose of their study was 13.1 mSv, while our low-dose protocol resulted in much low dose than that study. It has been reported that excessive dependence on CT is expensive and exposes the patients to nephrotoxic contrast media and ionizing radiation [41–43], making dose reduction desirable. Thus, it is of paramount importance to implement low-dose CTA protocol in aortic imaging. Although ultrasound is increasingly used to follow-up endovascular repair of aortic aneurysm as it does not involve ionizing radiation [44–46], CTA still remains the preferred method in the current clinical practice.

### Limitations

This study has several limitations. First, this study involved a relatively small number of patients (only 50 per group). Although no significant differences in objective or subjective image quality were detected, these results should be confirmed in a larger clinical cohort. Second, the diagnostic accuracy to detect aortic disease was not evaluated. The aim of the study was to assess the performance of CMs with different iodine doses in terms of image quality alone, not diagnostic accuracy. Third, only a single IR level was used in the low-iodine group. Further evaluation of different IR strength levels within each group should be performed. Lastly, we did not compare the reconstruction time between IR and conventional FBP. IR requires more time than a standard FBP reconstruction, which may influence the clinical utility.

## Conclusions

High-pitch, low-kilovoltage dual-source CT angiography is feasible in patients with suspected aortic diseases. Iterative reconstruction with SAFIRE appears to complement low-iodine CTA protocol acquired using a low voltage and high pitch technique with resultant 34% reduction in radiation dose, but with significant improvements in image quality.
